# Comment on: Evaluation of adjunctive mycophenolate for large vessel giant cell arteritis

**DOI:** 10.1093/rap/rkab011

**Published:** 2021-02-26

**Authors:** Rifat Mazumder, Chetan Mukhtyar

**Affiliations:** Department of Rheumatology, Norfolk & Norwich University Hospital, NHS Foundation Trust, Norwich, UK

Dear Editor, We have read the paper by Karabayas *et al.* [1] with great interest. The authors should be commended on their work for introducing a new drug, besides glucocorticoids, to the argument about the role of immunosuppression. As the authors hope, we too feel that this work will lead to a formal clinical trial for the role of MMF for the treatment of GCA.

GCA is large vessel vasculitis, and we think that the authors might be indicating involvement of the immediate branches of the aorta, including subclavian and axillary arterial involvement, when they term their subset as having ‘large vessel GCA’; a tautology, because there is no other type of GCA. The authors have made an elegant biological argument for considering mycophenolic acid derivatives, but we would be grateful for their thoughts on the following points.

1. Do the authors feel that involvement of different clusters of arteries is beyond being a phenotype of disease? If they make an argument for separate clinical trials for this phenotype, is it because they feel that this is not only a phenotypical difference, but also a mechanistic one? In that case, when a patient with GCA has both cranial and aortic branch involvement should we classify them as suffering from two diseases that might require different approaches?
2. GCA is a disease of the (relatively) elderly, often with multiple co-morbidities and risk of polypharmacy. The selection of medication for managing GCA without any published evidence might have needed negotiations regionally or nationally to allow the use of potent immunosuppressive agents, such as mycophenolic acid derivatives, outside of a clinical trial, especially in view of its expense before generic products were available in 2011 [2]. We would be grateful for any insight that the authors will have gained from that process to assist the British Rheumatology community negotiations with similar perennial discussions.
3. On a related note, there is published evidence and international consensus for the use of MTX in GCA [3–5]. We would value the thoughts of the authors on where they would put MTX in the management of their patients in the context of the findings of their paper.
4. For a population that was not supposed to have cranial involvement, the authors report a large number of patients who had cranial symptoms (62% had headache and 24% had visual symptoms). Are they satisfied that cranial involvement was satisfactorily ruled out without resort to temporal artery biopsy or ultrasonography?
5. Do the authors plan to extend their use to patients who might also have involvement of cranial arteries?

Once again, we thank the authors for sharing their work with this journal and its open access platform that allows for dissemination of scientific information freely. We look forward to their reply.

*Funding*: No specific funding was received from any bodies in the public, commercial or not-for-profit sectors to carry out the work described in this manuscript.

*Disclosure statement*: The authors have declared no conflicts of interest.
